# A Gut Feeling in Amyotrophic Lateral Sclerosis: Microbiome of Mice and Men

**DOI:** 10.3389/fcimb.2022.839526

**Published:** 2022-03-11

**Authors:** Sarah Martin, Carolina Battistini, Jun Sun

**Affiliations:** ^1^ Division of Gastroenterology and Hepatology, Department of Medicine, University of Illinois at Chicago, Chicago, IL, United States; ^2^ Department of Microbiology and Immunology, University of Illinois at Chicago, Chicago, IL, United States; ^3^ University of Illinois at Chicago (UIC) Cancer Center, University of Illinois at Chicago, Chicago, IL, United States; ^4^ Jesse Brown VA Medical Center, Chicago, IL, United States

**Keywords:** amyotrophic lateral sclerosis (ALS), gastrointestinal digestion, enteric neuron, metabolite, microbiome, bacteria, CNS - central nervous system, motor neurodegeneration

## Abstract

Amyotrophic lateral sclerosis (ALS) is a severely debilitating disease characterized by progressive degeneration of motor neurons. ALS etiology and pathophysiology are not well understood. It could be the consequences of complex interactions among host factors, microbiome, and the environmental factors. Recent data suggest the novel roles of intestinal dysfunction and microbiota in ALS etiology and progression. Although microbiome may indeed play a critical role in ALS pathogenesis, studies implicating innate immunity and intestinal changes in early disease pathology are limited. The gastrointestinal symptoms in the ALS patients before their diagnosis are largely ignored in the current medical practice. This review aims to explore existing evidence of gastrointestinal symptoms and progress of microbiome in ALS pathogenesis from human and animal studies. We discuss dietary, metabolites, and possible therapeutic approaches by targeting intestinal function and microbiome. Finally, we evaluate existing evidence and identify gaps in the knowledge for future directions in ALS. It is essential to understanding the microbiome and intestinal pathogenesis that determine when, where, and whether microbiome and metabolites critical to ALS progression. These studies will help us to develop more accurate diagnosis and better treatment not only for this challenging disease, but also for other neurodegenerative diseases.

## Introduction

ALS is a highly fatal neuromuscular disease currently with no cure. ALS has a median incidence of about 2.8 cases per 100,000 persons per year and a median prevalence about 5.4 cases per 100,000 persons for a median age at 61.8 ± 3.8 years (Chio et al., 2013). Although relatively rare, this public health impact of ALS is significant due to its morbidity and mortality.

The causes of ALS are largely unknown, only 10–15% of ALS cases are familial amyotrophic lateral sclerosis (fALS), with the remainder considered sporadic ALS (sALS) (Turner et al., 2017). Monogenic mutations in over 30 genes are associated with about ALS cases, including C9orf72, SOD1, FUS, and TARDBP/TDP43 (Nicolas et al., 2018; Volk et al., 2018). However, these monogenic forms explain only 15% of sporadic cases and 66% of familial cases (Turner et al., 2017). ALS pathogenesis is multifactorial and involves complex host-environment interactions. The risk factors include nutritional status, lower body mass index (BMI), smoking, high level of physical fitness, and military service (Wang et al., 2017). Environmental neurotoxins might contribute to ALS, e.g. β-Methylamino-L-alanine (BMAA) in people in Guam (Banack and Cox, 2003; Murch et al., 2004) and formaldehyde in factory workers (Weisskopf et al., 2009; Roberts et al., 2016).

Although ALS is a neurodegenerative disorder, progressive loss of motor neurons. Little is known about the gastrointestinal (GI) changes in ALS patients before and after the diagnosis, although autonomic dysfunction is reported in ALS. On one hand, in the literature, we see the comments that ALS patients do not complain of gastrointestinal symptoms and there is no GI symptom in ALS. On the other hand, there are cases that celiac disease with neurologic manifestations was misdiagnosed as ALS (Turner et al., 2007; Brown et al., 2010; Ham et al., 2017). There is report that gastrointestinal motor dysfunction occurred in ALS with delayed gastric emptying and delayed colonic transit times in patients (Wingate, 1999). Pancreatic and parotid deficiencies were observed in ALS patients (Charchaflie et al., 1974). There is a possible link between ALS and sensitivity to gluten based on an Israel study (Gadoth et al., 2015). In certain cases, an ALS syndrome might be associated with autoimmunity and gluten sensitivity. These reports suggest that ALS patients show GI symptoms, maybe at the early stage of their disease or before their diagnoses.

Growing evidence indicates that gut microbiome may actively contribute to ALS pathogenesis. ‘Microbiome’ includes all the microorganisms living (e.g., bacteria, virus, fungi) in the body. The definition of microbiome proposed by Whipps et al. is a broad one which describes microbiome as a “characteristic microbial community” in a “reasonably well-defined habitat which has distinct physio-chemical properties” as their “theatre of activity” (Whipps et al., 1988). Microbes inhabit the human body mediating key metabolic, physiological, and immune functions (Baquero and Nombela, 2012) and can be considered as another human organ. The last decade has witnessed a rebirth in interest in the microbes because of their novel and potential roles in disease pathogenesis and treatment. We are the first to report the elevated intestinal inflammation, reduced beneficial bacteria, and shift of microbiome profile in ALS (Wu et al., 2015; Rowin et al., 2017; Zhang et al., 2017; Zhang et al., 2021). Later, studies in human ALS and experimental animal models also reported the altered microbiome in the ALS (Fang, 2016; Labarre et al., 2017; Blacher et al., 2019; Figueroa-Romero et al., 2019; Burberry et al., 2020). Antibiotic usage, especially repeated use of antibiotics, are reported to be associated with a higher subsequent risk of ALS (Sun et al., 2019). The novel impact of gut microbiota on ALS development could explain some of the disease features beyond the influence of the genetic factor. However, microbiome studies in ALS are still very limited.

In the current review, we summarize the altered GI functions and roles of the gut microbiota in ALS, based on human data and experimental models. We discuss possible therapeutic approaches by targeting intestinal function and microbiome. Our goal is to evaluate existing evidence and identify gaps in the knowledge for future directions in ALS and other neurodegenerative diseases.

## Gastrointestinal (GI) Disorders in Human ALS

Pancreatic function was studied in ALS patients. In a 1967 study (Quick and Greer, 1967) that examined the pancreatic function of 5 ALS patients and 5 patients with non-ALS neurological diseases, they found that all 5 ALS patients displayed elevations in leucine aminopeptidase. 4 of the 5 ALS patients had abnormal results to tolbutamide testing. In the 4 ALS patients that underwent secretin testing, there were abnormalities in the response the secretin. The results of this study suggested that there is a disturbance of the function of the exocrine gland in patients with ALS, a finding that was consistent across patients. This is believed to be the first study that observed changes with this gland, as previous studies had only observed alterations of the endocrine gland. This study raised the question if there is a direct relationship between ALS and the pancreas or if alterations of the pancreas are due to secondary complications.

Utterback et al. (1970) analyzed the pancreatic function of 10 ALS patients and 1 patient with chronic pancreatitis. They found that the patients experienced a variety of intestinal symptoms. One patient experienced small bowel abnormalities which consisted of segmentation and small bowel coarsening. Another patient underwent rapid transit time of the small bowel. A third patient experienced small sliding hernia while another had a potential duodenal ulcer. Yet another patient had varices and a traction diverticulum of the esophagus. The authors determined that the only system with abnormalities was endocrine function. They concluded that abnormalities with glucose function resulted in malnutrition, diminished physical activity, and decreased muscle mass. In another study looking at the pancreatic function of ALS patients, Charchaflie et al. (1974), found that ALS patients experienced an insufficient pancreatic exocrine response. Additionally, there was no pathological structural changes observed, specifically organic lesions. The ALS patients experienced a marked decrease in amylase concentration. Post stimulation with citric acid, the parotid secretory response was significantly lower in the ALS patients, compared to the control group (Charchaflie et al., 1974). Additionally, the bicarbonate concentration in the saliva was significantly lower in the ALS patients. There was no difference in plasma osmolarity between ALS patients and normal controls. These results suggest a functional impairment of the exocrine gland. The pancreatic deficiencies were potentially a result of modifications of neuroendocrine mechanisms. They also raised the question whether ALS pathogenesis could be a result of digestive gland functional insufficiencies. However, there is a report on no abnormal pancreatic, but abnormal in glucose utilization in ALS. The authors believed host factors (e.g., age, malnutrition, diminished physical activity, and decreased muscle mass) may explain the observed abnormal glucose utilization.

Wingate (1999) reported GI motor dysfunction with delayed gastric emptying and colonic transit times in patients with ALS (Wingate, 1999). Nubling et al. investigated disease severity, intestinal dysfunction, and lower urinary tract symptoms (LUTS) in 43 patients with ALS (Nubling et al., 2014). Urinary incontinence was increased in patients with ALS aged ≥ 60 years compared to the EPIC cohort. Intake of muscle relaxants and anticholinergics was associated with both urinary incontinence and severity of symptoms (Nubling et al., 2014). Furthermore, a high prevalence of constipation (46%), but stool incontinence was only reported in 9% of the group. Overall, the increased prevalence of urge incontinence and high GI symptom burden imply in patients with ALS. There are cases that celiac disease with neurologic manifestations was misdiagnosed as ALS (Turner et al., 2007; Brown et al., 2010; Ham et al., 2017). An ALS syndrome may be associated with autoimmunity and gluten sensitivity (Group, 2016).

In 1987, Nakano et al., found an increase in the size of pale hepatocytes, mild fatty infiltration, and variability in the nuclear size of these hepatocytes in ALS patients (Nakano et al., 1987). Additionally, fibrosis around the hepatocytes was present, as well as abnormalities of the hepatocellular mitochondria. This was one of the first studies to observe this fibrosis and was found in 76.2% of ALS patients versus 42.3% of controls. However, there was no relationship between these abnormal findings in the livers of ALS patients with the progression of the disease. This study suggest that abnormalities of the liver potentially occur in ALS and that the presence of these abnormalities would not be secondary to disease symptoms.

Jonsson et al., 2008 looked at the liver pathology from a single ALS patient in a case report. They found granular mutant SOD1 inclusions in the hepatocytes and kidney tubular epithelium and the absolute levels of G127X SOD1 were greater in the liver than in the temporal lobe. Staining of the hepatocytes was more widespread and uneven. The liver had inactive micronodular cirrhosis with some steatosis. Blood plasma levels indicated a moderate form of liver disease. From these results, the authors asked the question whether G127X SOD1 could contribute to these alterations to the liver.

The enteric nervous system (ENS) provides neural control in the gastrointestinal tract. When analyzing human ALS patient tissue, Luesma et al., 2014 (Luesma et al., 2014) found that there was evidence of the presence of adult enteric neurons stained with c-Ret (a RET kinase inhibitor) in the myenteric and submucosal ganglia of the adult duodenum. It suggested that c-Ret was necessary for the human mature ENS. Further studies would be needed to look at this connection with neurodegenerative diseases such as ALS.

Our study (Rowin et al., 2017) evaluated infection and markers of intestinal inflammation and the human gut microbiome in stool samples from ALS patients. A majority of patients had signs of intestinal inflammation. This is the first comprehensive examination of inflammatory markers in the stool of ALS patients. A previous study (Zhang et al., 2009) reported that the level of plasma lipopolysaccharides (LPS), a bacterial endotoxin, significant increased and had a positive correlation with activation of blood monocyte/macrophage in sALS groups, and LPS was most elevated in patients with advanced sALS disease (Zhang et al., 2009). Circulating endotoxin and systemic immune activation in sALS suggested intestinal leakage and local inflammation in these sALS patients.

Clearly, there are GI disorders in patients with ALS. We summarized the current literature in **Table 1**. These reports suggest that ALS patients show GI symptoms, maybe at the early stage of their disease or before their diagnoses. However, GI symptoms for pre-diagnosis of ALS are largely ignored. It is challenging to perform systemic studies before the diagnoses.

**Table 1:** Involved organs of GI tract in patients with ALS.

Organs	Symptoms	Stage of disease	Sample size	References
Pancreas and parotid glands	Parotid flow rate and bicarbonate concentration from ALS patients were found to be decreased by about 66% and 70% respectively, when compared with controls.		17 patients	https://pubmed.ncbi.nlm.nih.gov/4852110/
Pancreas		Symptomatic	5 patients with ALS and 5 additional patients with neurologic disease other than ALS	https://pubmed.ncbi.nlm.nih.gov/6066871/
Pancreas/Small Intestine	There were varices and a traction diverticulum of esophagus in one patient, a small sliding hernia in another, and a possible duodenal ulcer. There was some segmentation and coarsening of the small bowel in one patient and a rapid transit time in another.	Symptomatic	10 patients with ALS and 1 patient with chronic pancreatitis	https://pubmed.ncbi.nlm.nih.gov/5505684/
ENS	c-Ret immunoreactivity in myenteric and submucosal ganglia, GFAP-ir labeled cells appeared within myenteric ganglia while c-Kit labelled cells appeared around myenteric ganglia. Propose the ENS marker for the ALS patients		12 human sample No direct data from ALS patients	https://pubmed.ncbi.nlm.nih.gov/24868525/
Intestine	Intestinal inflammatory markers in stool samples	Symptomatic	5 patients with ALS	(Rowin et al., 2017) https://pubmed.ncbi.nlm.nih.gov/28947596/
GI motor dysfunction	delayed gastric emptying and colonic transit times	Symptomatic		(Wingate, 1999)
GI motor dysfunction/urinary tract	investigated disease severity, lower urinary tract symptoms (LUTS) and intestinal dysfunction.	Symptomatic	43 patients with ALS	(Nubling et al., 2014)
Liver	Granular G127X SOD1 inclusions were seen in hepatocytes in the liver, and the staining was more widespread. Granular G127X SOD1 inclusions were also found in kidney tubular epithelium. There was presence of inactive micronodular cirrhosis with some steatosis of the liver. There were some elevated liver disease markers.	Postmortem	1 patient suffered from rapidly progressing ALS and died of respiratory insufficiency and 5 age-matched patients who died of non-neurological diseases	https://pubmed.ncbi.nlm.nih.gov/18409196/
Liver	Increased size of pale hepatocytes, mild fatty infiltration	Symptomatic	21 patients with ALS and control specimens randomly chosen from 215 patients with various liver diseases	https://pubmed.ncbi.nlm.nih.gov/3800708/
Systemic	Increased LPS, Inflammation	Symptomatic	sALS	(Zhang et al., 2009)

## Gut Pathophysiology in ALS Mouse Models

Transgenic animals are expected to imitate the key features of ALS. There are different ALS experimental models, with their advantages and disadvantages (Philips and Rothstein, 2015). In patients with ALS, there are spinal cord and muscle pathology. About 10% of familial ALS cases are attributed to mutations in Cu, Zn superoxide dismutase (SOD). Normal SOD catalyzes the dismutation of superoxide radicals into hydrogen peroxide and molecular oxygen. The ALS pathology can be modeled in hSOD1^G93A^ mice, which carry the human point mutant SOD1 ^G93A^.

Similar to patients with ALS, the hSOD1^G93A^ mice showed liver problems, e.g., hepatic abnormalities and lymphocytic infiltration (Lee and Yang, 2018). Lee and Yang (2018) investigated the liver of hSOD1^G93A^ mice transgenic mice, changes in liver protein expression over the course of disease progression were found. This included a significant increase in inflammatory cytokines (TNF-α, IL-1β, and CD11b), oxidative stress-related proteins (NQ01 and ferritin), and regulators of cell death (Bax and pJNK). For pre-symptomatic mice hSOD1^G93A^ mice, there was a significant increase in fibrosis-related proteins (HDAC4, GADD45α, and PDGFb) in the liver. These findings suggested that liver dysfunction may lead to liver fibrosis in hSOD1^G93A^ mice. Additionally, liver dysfunction in hSOD1^G93A^ mice could potentially be mediated by increased inflammation and oxidative stress, in addition to the upregulation of fibrosis-related proteins.

We found the damaged tight junctions (TJs) and increased intestinal permeability in the hSOD1^G93A^ mice (Wu et al., 2015). TJs act as barriers in the intestinal epithelial cells to protect host from pathogens, inflammation, and other injuries (Zhang et al., 2013; Zhang et al., 2019). We studied hSOD1^G93A^ and age matched wild type mice and found that TJ ZO-1 protein expression was significantly decreased in the intestine of G93A, compared to WT mice at 2-month-old. The G93A mice do not show ALS symptoms until 3 months old. The adherent junction protein E-cadherin was significantly decreased in the intestines of G93A mice, compared to the age-matched WT mice. ZO-1 was a tight junction protein restricted to cellular borders in a smooth and organized pattern at the apical side of the healthy colons. We identified abnormal and disrupted ZO-1 distribution in the membrane of intestinal epithelial cells in ALS mice at the age of 2 months. We also showed a significant increase in abnormal Paneth cells and increased inflammatory cytokine IL-17 in hSOD1^G93A^ mice (Wu et al., 2015). As the changes in intestine function occurs early, it suggests the possibility that altered intestinal homeostasis may take place before the decline of neuromuscular function in ALS mice. This study demonstrated that impaired gut-neuromuscular crosstalk could potentially contribute to the progression of ALS.

Intestinal mobility is a key physiologic parameter governing digestion and absorption of nutrients affected by the ENS, microbiome, and host genetics (Rao and Gershon, 2016; Niesler et al., 2021). Few studies in general have been conducted looking at the connection between the ENS in the SOD1G39A ALS model. Our recent study (Zhang et al., 2021) reported the dysfunction of ENS in SOD1^G39A^. Before ALS onset, 2-month-old G93A mice had significant lower intestinal motility, decreased grip strength, and reduced time in the rotarod. We observed increased GFAP, a marker for ENS, and decreased SMMHC expression, a marker for smooth muscle (Zhang et al., 2021). These ENS changes are significantly correlated with increased aggregation of hSOD1^G93A^ in the spinal cord, small intestine, and colon (Zhang et al., 2021).

In a 2011 study by Finkelstein et al, the authors discovered that the liver parenchyma underwent significant atrophy and lipid redistribution, while an increase in natural killer T cells in both the lymphoid organs and spinal cord of the mSOD1 mice compared to WT mice was also observed. While the liver itself decreased in size as the disease progressed, the proportions of natural killer T cells in the liver increased by 4-fold at the end-stage of the disease. In mSOD1 mice with the C57B1 background, the natural killer T cells had a similar fate. T cells were significantly enhanced in the spinal cord and spleen of mSOD1 mice. Additionally, natural killer cells were significantly enhanced in the spinal cord of mSOD1 mice, but not in the spleen and liver. This study showed that changes to liver are present during disease progression in transgenic mice and therapeutic interventions that target these changes are needed.

Some animal models may not always exhibit similar co-morbidity phenotypes to human patients with ALS. The transgenic mice overexpressing a mutant human TDP43 gene (Hatzipetros et al., 2014), the Prp-TDP43^A315T^ mice, exhibited decreased survival rates that may be associated with neurodegeneration, occurring in the myenteric plexus of the colon. In these Prp-TDP43A315T mice, death coincided with severe GI pathology. In the lower GI tract, distal to the cecum, there were pathological changes, such as swelling, intraintestinal coagulated blood, and necrotic tissue. It is most likely a result of overexpression of the transgene in the lower GI tract, as opposed to the upper GI where there is no expression of the transgene and no neurodegeneration. The neurodegeneration of the lower GI tract is a feature that is unique to the Prp-TDP43^A315T^ mice. Similar findings have been reported by others (Guo et al., 2012). TDP43 ^A315T^ mice exhibited not only a reduction in food ingestion and defecation, but also a thinned colon and swollen small intestine. The swollen small intestine and thinned large bowel also indicate peristaltic malfunction in the TDP43 ^A315T^ mice. In the colon, the accumulated TDP43 ^A315T^ in the myenteric nerve plexus and myenteric neuron degeneration could result in phenotypes similar to hypoganglionosis. These results, in addition to no limb paralysis present at the end stage of disease progression, suggested that intestinal dysfunction contributed more to their death than severe muscle weakness in the TDP43 ^A315T^ mice. The authors believed that while the TDP43 ^A315T^ model can be used to analyze GI tract degeneration, it did not display a phenotype that was acceptable to study ALS therapeutics.

Herdewyn et al. (2014) found a significant reduction in the ability of the transgenic TDP43 ^A315T^ mice to generate propulsive contractions. There was degeneration of nitric oxide synthase (NOS) (Fleck et al., 2021) neurons in the enteric nervous system, but no difference in choligeneric neurons. Because NOS and excitatory neurons both regulate intestinal peristalsis, lack of both excitation and inhibition would result in a failure to generate contractile force and the prevention of peristalsis. The degenerated NOS neurons suggested the loss of inhibitory control that led to abnormal intestinal propulsion, dysmotility and pseudo-obstruction, and sudden death (Herdewyn et al., 2014). There was downregulation of endogenous TDP-43 in the spinal cord and brain of the TDP43 ^A315T^ mice prior to the observation of neurodegeneration. The feeding of jellified food prevented intestinal obstruction, allowing the motor phenotype to develop, and significantly extending survival. These results suggested that the degeneration of NOS neurons in the myenteric plexus resulted in intestinal dysmotility in the TDP43 ^A315T^ mice. In yet another study using the TDP43 ^A315T^ model (Esmaeili et al., 2013), the authors found intestinal dilation with the caecum and lower ileum mildly to moderately dilated and contained dry, firm fecal material. One animal had a fibrous band at the ileocecocolic junction. The stomachs of the animals were all empty. The authors concluded that the animals were dying due to a reduction in motility that originates in the ileocaecal area of the GI tract. A limitation of this study is a general limit of the TDP43 ^A315T^ animal model itself –lacking lower motor neuron degeneration in the ventral horn of the spinal cord. Toxicity from TDP43 overexpression may lead to a reduction in GI motility, though it is still not fully understood how TDP-43 overexpression leads to GI tract abnormalities in the TDP43 ^A315T^ mice. These studies suggest that gastrointestinal complications may have been the cause of death for TDP43 ^A315T^ mice and not spinal motor neuron loss.

Studies in the animal models have shown the GI petrophysical changes before the motor neuron degeneration was observed. We summarized these findings in **Table 2**. Although the transgenic animal models cannot sufficiently represent the development of human ALS, especially the sALS, studies in these models still provide valuable data and mechanisms during the disease progression.

**Table 2:** Involved organs of GI tract in ALS animal models.

Organs	Symptoms	Stage of disease	Sample size	References
Colon	There was swelling, intraintestinal coagulated blood and necrotic tissue in the lower GI tract, distal to the cecum, versus no pathology in age-matched wild-type mice. The slowing down of gut motility was significantly different in TDP43 mice compared to WT mice.	Presymptomatic and symptomatic	Over 650 transgenic C57BL/6J Prp-TDP43A315T male and female mice	https://pubmed.ncbi.nlm.nih.gov/24141148/
Colon	Transgenic mice experienced gradually reduced food ingestion and defecation compared to WT controls. They also found thinned colon and swollen small intestine in the TDP-43 A315T mice.	Presymptomatic and symptomatic	12 transgenic mice with the TDP43A315T mutation and 12 non-transgenic litter mates	https://pubmed.ncbi.nlm.nih.gov/22578468/
Intestine	Intestinal dilation, mildly dilated cecum and lower ileum, contained dry firm fecal material, jejunum and duodenum contained watery material, stomachs were empty, compared to age-matched wild-type controls.	Prior to disease development	15 male, 15 female, TDP43A315T transgenic mice 15 non-transgenic control	https://pubmed.ncbi.nlm.nih.gov/23317354/
ENS/Colon/ Small Intestine/Cecum	Degeneration of NOS neurons in the ENS and a high transgene expression of human mutant TDP-43 in the nuclei of neurons of the myenteric plexus. Mice had an extremely rigid abdomen (intestinal obstruction), a thinned colon, enlarged cecum and distension of the small intestines. For the degeneration of NOS neurons, the number of neurons was decreased and the neurons that remained were enlarged. Nitric oxide synthase expressing neurons were severely affected in terminal ileum and colon.	Presymptomatic and symptomatic	Numbers vary for each comparison. TDP43A315T) mice and NTG C57BL/6 J mice	https://pubmed.ncbi.nlm.nih.gov/24938805/
Colon/ Small Intestine	LPS, Inflammatory cytokines, leaky gut	Presymptomatic and symptomatic	Wild-type and hSOD1 ^G93A^ mice, from 3-15 mice per comparison	(Wu et al., 2015)
Colon/ Small Intestine	Butyrate treatment to reduce the GI symptoms	Presymptomatic and symptomatic	Wild-type and hSOD1 ^G93A^ mice, from 3-15 mice per comparison	(Zhang et al., 2017)
ENS/Colon/ Small Intestine	Slower bowel movement, abnormal ENS	Presymptomatic and symptomatic	Wild-type and mSOD1 ^G93A^ mice, from 6-20 mice per comparison	(Zhang et al., 2021)
Metabolites/ Inflammation/inter-organ communications	Changes in carbohydrate levels, amino acid metabolism, and formation of gamma-glutamyl amino acids in the ALS mice. Shifts in several microbially-contributed catabolites of aromatic amino acids agree with butyrate-induced changes in composition of gut microbiome. Butyrate treatment significantly suppressed the IBA1 level in the microglia of SOD1^G93A^ mice. The serum IL-17 and LPS were significantly reduced in the butyrate treated SOD1^G93A^ mice.	Presymptomatic and symptomatic	Wild-type and mSOD1 ^G93A^ mice with or without butyrate treatment, untargeted and target metabolomic studies	https://biorxiv.org/cgi/content/short/2022.01.15.476456v1
Liver	The liver had the most prominent increase in the proportion of NKT cells compared to WT mice. The liver decreased in size as the disease progressed. T cell abundance was significantly lover in mSOD1 relative to WT. There was also atrophy of hepatocytes and lipid aggregation in the liver. Liver parenchyma also had significant atrophy.	Pre-symptomatic and symptomatic	Wild-type and mSOD1 ^G93A^ mice, anywhere from 3-14 mice per comparison	https://pubmed.ncbi.nlm.nih.gov/21829620/
Liver	Increase in inflammatory expression (TNF-alpha and IL-1B), increase in oxidative stress-related protein (NQO1 and ferritin), fibrosis-related proteins are upregulated (HDAC4, GADD45α and PDGFβ)	Presymptomatic and symptomatic	non-transgenic, pre-symptomatic (2-month-old) transgenic mice, symptomatic (4-month-old) transgenic mice	https://pubmed.ncbi.nlm.nih.gov/30130789/

## Altered Gut Microbiome in ALS Mouse Models

There are known active host-microbiome interactions in the GI tract. Our 2015 study has demonstrated the altered bacterial abundance, reduced levels of butyrate-producing bacteria in the ALS SOD1^G93A^ animals (Wu et al., 2015). The intestinal microbiome was altered as butyrate producing bacteria (e.g., *Butyrivibrio Fibrisolvens*) were reduced in ALS mice at the age of 2 months before the onset of disease. The cellular and physiological changes mirror the population and function changes in the gut microbiome of the SOD1^G93A^ mice. With an interventional study attempting to alleviate symptoms through treatment with sodium butyrate (Zhang et al., 2017). SOD1G93A mice chronically treated with sodium butyrate fared better than those not given the compound, showing improved intestinal barrier function and delays in both weight loss and death of 50 and 38 days respectively. Further longitudinal studies (Zhang et al., 2021) demonstrated the early stage microbial changes in the mice at 1-month old. The altered bacterial community is related to autoimmunity *(e.g., Clostridium* sp. *ASF502, Lachnospiraceae bacterium A4)*, inflammation (e.g., *Enterohabdus Muris*), and metabolism (e.g., *Desulfovibrio fairfieldensis*) at 1- and 2-month-old SOD1^G93A^ mice, suggesting the early microbial contribution to the ALS pathological changes (Zhang et al., 2021).

A 2019 study (Blacher et al., 2019) confirmed the significant differences in the fecal microbiome composition of the SOD1^G93A^ mice, compared with wild-type littermate controls. In the SOD1-Tg mice (generally known as SOD1^G93A^ mice), antibiotic treatment was associated with a significant exacerbation of motor abnormalities in the mice throughout the progression of the disease. Motor neuron cell death increased after chronic exposure to antibiotics. In antibiotic treated SOD1^G93A^ mice, the addition of *P. distastonis* and *R. torques* exacerbated disease progression. Treatment with *L. gasseri* and *P. melaninogenica* displayed disease promoting effects in a some but not all behavioral tests. None of the 11 bacterial strains that were tested affected motor abilities in the wild type mice. There was a slow reduction in the levels of *A. muciniphila* in SOD1^G93A^ mice with progression of the disease, while its levels remained constant across time in the wild-type mice. *A. muciniphila* treatment significantly and substantially prolonged the lifespan of SOD1^G93A^ mice, compared to vehicle-treated mice or SOD1^G93A^ mice treated with other commensal microbiome species. *A. muciniphila* favorably modulated the course and severity of the disease in mice, whereas *P. distastonis*, *R. torques*, and possibly *L. gasseri*, *P. melaninogenica* adversely modulated the course and severity of ALS in mice. Overall, these results confirm the involvement of the gut microbiome in SOD1^G93A^ mice.

Another study detected differences in the gut microbiome between SOD1^G93A^ mice and controls at multiple time points (Figueroa-Romero et al., 2019). Interestingly, they noted that the ALS phenotype in the mice, including survival, varied depending on the animal facility used to house the mice; the authors suggested this curious observation was a result of differences in gut microbiota (Figueroa-Romero et al., 2019).

Intestine acts as a barrier to harmful molecules. A range of dietary toxins has been implicated in the cause of ALS, and dysbiosis could facilitate their entry because dysbiosis leads to increased intestinal permeability, as we observed in the previous report (Wu et al., 2015).

The ALS C9orf72 mouse model harbors loss of function mutations in the orthologous gene C9orf72 (Philips and Rothstein, 2015). A 2020 study (Burberry et al., 2020) found that the murine norovirus, *Helicobacter* spp., *Pasteurella pneumotropica* and *Tritrichomonas muris* were significantly more abundant in *C9orf72^Harvard^* mice than in C9orf72^Broad^ mice. The increase in *Helicobacter* spp. levels suggested that gut microbiota changes may be the underlying mechanism of increased rate of mortality and inflammatory phenotypes in *C9orf72^Harvard^* mice. Treatment with antibiotics significantly decreased both the abundance and diversity of bacterial species without affecting the levels of murine norovirus, compared to vehicle treated controls. The bacterial species that decreased included *Helicobacter* spp. Administering lifetime antibiotics to the C9orf72^Harvard^ -/- resulted in the complete suppression of inflammatory or autoimmune phenotypes, suggesting that when C9orf72 function is decreased, gut bacteria signals and promote autoimmunity and inflammation. Acute administration of broad-spectrum antibiotics reduced inflammatory and autoimmune phenotypes and improved rotarod performance in the mutant mice.

We summarized the existing studies on gut microbiome in ALS models in **Table 3**. Different ALS experimental models show altered microbiome, and the pathophysiological changes could by modulated by bacterial metabolites or antibiotic treatment. These results suggested that gut microbiota and environment modify immunity, neuroinflammation, and motor deficits in the development of ALS.

**Table 3:** Microbiome studies in ALS experimental models.

Study	Key Bacterial changes	Sample size	Methods	Reference
Leaky intestine and impaired microbiome in SOD1 ^G93A^	Reduced butyrate-producing bacteria (*Butyrivibrio Fibriosolvens*), *E. coli*, and *Fermicus* in ALS mice at the age of 2 months	SOD1 ^G93A^ mice and age-matched wild-type mice	Western blot analysis, immunofluorescence, ELISA, real-time qPCR, fetal microbiome sequencing	https://pubmed.ncbi.nlm.nih.gov/25847918/
Target Intestinal microbiota to alleviate disease progression in ALS	Butyrate treatment significantly increased *Butyrivibrio* species, also significantly increased *Bacteroide*, *Odoribacter*, *Eubacterium*, significantly depleted *Tannerella*	SOD1 ^G93A^ and age-matched wild-type mice	Western blot analysis, immunofluorescence, cell transfection and live cell imaging, real-time qPCR	https://pubmed.ncbi.nlm.nih.gov/28129947/
Effects of Intraoperative Vagal Nerve Stimulation (VNS) on the microbiome in SOD1 ^G93A^ mice	In all mice, the most abundant bacteria were in the *Bacteroidales* group and the bacterial families of *Rikenellaceae* and *Lachnospiraceae.* VNS did not change the microbiome in the SOD1 ^G39A^ mice.	30 SOD1^G93A^ mice and 30 age-matched WT controls	Vagal nerve surgical stimulation, fecal collection, DNA extraction, rRNA sequencing	https://pubmed.ncbi.nlm.nih.gov/30424824/
Microbiome, immune system and epigenome with disease progression in SOD1 ^G93A^ mice.	In the ileum, *Firmicutes* were more abundant than *Bacteroidetes* in SOD1^G93A^ mice versus WT mice. At 90 days, *Firmicutes* were less abundant than *Bacteroidetes* in SOD1 ^G93A^ mice versus WT mice. In feces, *Firmicutes* were more abundant in SOD1^G93A^ mice aged 37 and 60 days compared to WT mice. *Bacteroidetes* were more abundant in colon from the SOD1^G93A^ 60-day-old mice.	8 30-day-old SOD1^G93A^ mice and WT control littermates for each comparison	Motor function assessed using rotarod, collected fecal and intestinal content samples, isolated bacterial DNA for sequencing, blood, brain, spleen, and spinal cord leukocytes collected, bone marrow collected, flow cytometry	(Figueroa-Romero et al., 2019) https://pubmed.ncbi.nlm.nih.gov/31597644/
C9orf72 suppresses systemic and neural inflammation induced by bacteria	*Helicobacter*, *Pasteurella pneumotropica*, *Tritrichomonas muris* were significantly more common in C9orf72(Harvard) mice than in C9orf72(Broad) mice. *Helicobacter* were found in both pro-inflammatory environments but were not found in pro-survival environments.	Range between experiments from 10 to 114;C9orf72^+/+^, C9orf72^+/-^, C9orf72^-/-^ mice	Motor behavior assessed using rotarod, fecal pellets and intestinal contents collected, immunofluorescence, flow cytometry, PCR	https://pubmed.ncbi.nlm.nih.gov/32483373/
Longitudinal studies of ENS, SOD1^G93A^ aggregation, and microbiome modulation by butyrate or antibiotics	Butyrate or antibiotic treatment resulted in a significantly longer latency to fall in the rotarod test, reduced SOD1^G93A^ aggregation, and enhanced enteric neuromuscular function. Feces from 2-month-old SOD1^G93A^ mice significantly enhanced SOD1^G93A^ aggregation in human colonoids transfected with a SOD1^G93A^-GFP plasmid. Longitudinal studies of microbiome data showed the altered bacterial community related to autoimmunity *(e.g., Clostridium* sp. *ASF502, Lachnospiraceae bacterium A4)*, inflammation (e.g., *Enterohabdus Muris*), and metabolism (e.g., *Desulfovibrio fairfieldensis*) at 1- and 2-month-old SOD1^G93A^ mice, suggesting the early microbial contribution to the pathological changes.	Presymptomatic and symptomatic	Wild-type and mSOD1 ^G93A^ mice with butyrate or antibiotic treatment, from 6-20 mice per comparison Human colonoids transfected with a SOD1^G93A^-GFP plasmid	(Zhang et al., 2021)
Gut microbiome and metabolites in modulating ALS in mice	Addition of *E.coli* nadA-/- decreased hanging-wire grip test performance and worsened neurological score in SOD1 mice compared to WT mice. Adding *P. distasonis* and *R. torques* exacerbated progression of the disease. *A. muciniphila* improves motor neuron degeneration and increases lifespan in SOD1 mice. Mice given *A. muciniphila* accumulate nicotinamide in the central nervous system. Nicotinamide alone had a limited protective role to slow the disease progression. Facility-dependent changes of microbiome were found, suggesting that genetic susceptibility to ALS and a locally prevalent microbial signature drive early dysbiosis.	For mice, varies with experiment. Pooled results = ~10-30 mice	For mice, depleting the microbiome and quantifying motor abilities, DNA sequencing, mono-inoculating 11 strains of bacteria into mice, untargeted metabolomic profiling.	https://pubmed.ncbi.nlm.nih.gov/31330533/

## Human Studies of the Gut Microbiome in ALS

When examining 6 ALS patients and 5 healthy people without ALS, a 2016 study (Fang et al., 2016) found microbiomes of ALS showed a significant difference in the global bacterial gene content compared to healthy controls. Phylum *Bacteroidetes*, class *Bacteroidia*), order *Bacteriodales*, and genus *Dorea* were significantly higher in ALS patients than in the healthy controls. *Lachnospiraceae* (at family level), *Firmicutes* (at phylum level) and *Clostridia* (at class level), *Oscillibacter* (at genus level), *Family XII* (at family level), *Anaerostipes* (at genus level), *Lachnospiraceae* (at genus level), and *Clostridiales* (at order level) were significantly higher in the healthy controls than in the ALS patients. In the healthy controls, the 3 dominant phyla in 80% of the fecal microbiomes was *Bacteroidetes*, *Firmicutes*, and *Actinobacteria.* There was a significant decrease of *Oscillibacter*, *Anaerostipes*, and *Lachnospiraceae* in ALS patients.

The gastrointestinal complaints of the ALS patients predated neurological symptoms. In 2017 (Rowin et al., 2017), we studied the medical tests of 5 patients with ALS. The GI symptoms included gastroesophageal reflux, disease, chronic constipation, intermittent diarrhea, and abdominal pain and bloating. All ALS patients showed dysbiosis indicated by a decreased diversity of the microbiome compared to the healthy individuals. Patients 1, 3, and 5 had reduced benefit bacteria at the phylum level. Three patients (#1, 3 and 5) had a low Firmicutes/Bacteroidetes (F/B) ratio. Low *Ruminococcus* spp. occurred in all three ALS patients with a low F/B ratio, while *Clostridium* spp. and *Roseburia* spp. were low in patient 1. Patient 5 had high *Bacteroides- Prevotella*, *Odoribacter* spp., *Barnesiella* spp. and *Bacteroides vulgatus*. Patient 3 had high *Bacteroides vulgatus*, which could be the cause of a low F/B ratio. Patient 2 did not have a low F/B ratio, but the patient was taking probiotic supplements at the time of stool sample collection. All the ALS patients had a change to their gut microbiome that was characterized by a low diversity of the microbiome compared to the healthy individuals who had intact abundance within their gut microbiome. Our results suggested a potential role of intestinal inflammation and microbiome in the development and/or progression of human ALS.

In an observational study of 37 human ALS patients versus 29 healthy BMI- and aged- matched family members as controls (Blacher et al., 2019), the microbiome composition of ALS patients was significantly different from those of the healthy controls. There was only a small significant difference in the abundance of certain bacterial species in ALS patients. The microbiomes of the ALS patients showed a significant difference in the global bacterial gene content (Blacher et al., 2019). Additionally, there was a significant reduction in the abundance of several genes that map to the *Akkermansia muciniphila* genome.

A 2017 study (Brenner et al., 2018) analyzed the microbiome of 25 human patients with ALS and 32 healthy age- and gender- matched controls. They primarily found different proportions of uncultured *Ruminococcaceae*, However, aside from this finding and a difference in the overall number of microbial species, there was no significant differences in the quantity, diversity, and relative abundance of the fecal microbiota of the ALS patients. The range of different types of species of the intestinal microbiota did not differ between the ALS patients and the healthy controls. The F/B did not differ significantly between the ALS patients and healthy controls. The authors raised the possibility that while the gut microbiota of ALS patients experienced a change in metabolite production, ALS may not be associated with a significantly altered gut microbiota composition. This conclusion may be because the authors used a strict selection of ALS patient that had a high revised ALS functional rating scale without bulbar or respiratory symptoms, in order to avoid confounding factors. They analyzed a larger group of patients that were clinically well characterized. However, when ALS patients are at late stages of the disease, they may suffer from a variety of conditions that can affect the composition of the gut microbiota, influencing results. Revisiting this study, we actually know that F/B at the phylum level is a not realizable readout to indicate the functional changes of microbiome and the changes of microbiome should be investigated in-depth. The different proportion of Ruminococcaceae was an indicator of altered microbiome in these ALS patients. The authors only selected patients that were at a high functional level and did not have any overt bulbar or respiratory symptoms, therefore producing small and possibly inconsequential significant results. To generate more meaningful correlation, studies that involve all stages of the disease would need to be conducted. A secondary limitation is that altered metabolite production may be present in ALS patients, further disrupting the gut microbiota.

A 2018 study (Mazzini et al., 2018) analyzed the microbiota in 50 ALS patients and 50 healthy controls that were matched for sex, age, and origin. They found that there was a clear division between the bacterial profiles of ALS patients and the healthy controls. There was a lower DNA concentration in ALS patients compared to the controls. There was a low abundance of *Clostridium* and yeast, and there was a higher abundance of *E. coli* and *Enterobacteria*. The results of this study confirmed the ongoing hypothesis that the gut microbiota is altered in ALS patients and could potentially play a role in the pathogenesis of ALS.

In a 2020 study (Zeng et al., 2020) that examined the gut microbiota associated with ALS in 20 human patients and 20 age-matched healthy controls, there was a significant increase in the relative abundance of Bacteroidetes at the phylum level, *Kineothrix*, *Parabacteroides*, *Odoribacter*, *Sporobacter*, *Eisenbergiella*, *Mannheimia*, *Anaerotruncus*, and *unclassified Porphyromonadaceae* at the genus level and a significant reduction in *Firmicutes* at the phylum level and *Megamonas* at the genus level. Additionally, Bacteria at the domain level, Bacteroidetes at the phylum level, Bacteroidia at the class level, Bacteroidales at the order level, and Porphyromonadaceae at the family level were higher in ALS patients compared to controls. Microbes at the species level were significantly higher in ALS patients, including *Sulfuricurvum kujiense*, *Cyanothece* sp. and *Haladaptatus paucihalophilus*, while *Enterococcus columbae* was significantly decreased. These results indicated that the community diversity and species composition of the fecal microbiota is changed in patients with ALS. Limitations of this study include the fact that the authors could not determine whether the correlation between the intestinal dysbacteriosis and ALS occurred before or after the onset of ALS, so it still cannot be determined whether a causal relationship exists.

Another 2020 study (Di Gioia et al., 2020) examined 50 human patients with probable or defined sporadic and 50 age-matched case controls. The longitudinal study addressed the microbiota in patients with ALS and the impact of a probiotic supplementation on the gut microbiota and ALS progression. There was a significantly lower amount of *Clostridium* cluster I and yeasts and a higher concentration of *E. coli* in the ALS patients. A lower DNA concentration was present in the ALS patients, while the number of total bacteria was not significantly different between the ALS patients and the control group. *Enterobacteriaceae* were higher in ALS patients. The more representative phyla were *Bacteroidetes* and *Firmicutes* in both groups, which showed an abundance of 40-45%. *Cyanobacteria* phylum members were significantly higher in ALS patients versus control patients. At the genus level, *Lactobacillus*, Citrobacter, *Coprococcus*, and *Ruminococcaceae* were more abundant in the ALS patient group. *Veillonellaceae* and *Lachnospiraceae* families, *Parasutterella*, *Ruminococcus* and *Subdogranulum* were reduced in ALS group, compared to the controls. Overall, these results suggested the existence of differences in the bacterial composition between healthy controls and patients with ALS. This study demonstrated the imbalance of intestinal bacteria that may play important roles in modulating the central nervous system (CNS). Limitations of this study include the restricted group size - 20 ALS patients were not able to complete the study and 1 died. Additionally, intra-individual variability was not incorporated into the study.

In a 2021 study (Niccolai et al., 2021), they looked at the gut microbiota and its SCFA composition and the intestinal and systemic inflammatory response in ALS patients compared to their healthy controls. In 19 ALS patients and 9 healthy family caregivers that were matched for sex and were close in age, there were significant differences in cytokine levels. When looking at the cytokine levels in the serum, they found that IL-8, IL-15, MCP-1 and VEGF-A levels were significantly lower in ALS patients compared to controls. When looking at the cytokine levels in the fecal samples, they found that IL-2 was higher in ALS patients compared to controls, although the difference was not significant. Additionally, IL-21 was lower in ALS patients that had a faster progressing form of the disease, compared to patients that has medium or slow progressions. Patients with a slower progression of ALS had lower microbial diversity than other variations of progression. Similar results were displayed for ALS patients with the classical form of the disease compared to patients with various clinical phenotypes. Control samples displayed a higher Firmicutes/Bacteroides ratio than the ALS patients with the Firmicutes and Bacteroides being the most represented phyla in all samples. ALS patients that had a medium progression form of the disease displayed a higher Firmicutes/Bacteroides ratio than both ALS patients with other forms of progression and healthy controls. However, these differences did not appear to be statistically significant. At the genus level, the healthy controls displayed an enrichment of *Erysipelotrichaceae_UCG-003*, *Fusicatenibacter*, and *Subdoligranulum* compared to the ALS patients. There appeared to be an increase of Streptococcus in the samples from ALS patients with a slow progression of the disease. From this study, the difference in serum profile between the ALS patients and healthy controls suggests that there may be an intestinal inflammatory aspect to ALS. Since IL-21 was expression more in patients with a slow form of ALS compared to a fast progression, it could potentially be developed into a prognostic biomarker. The study confirmed that there is dysregulation occurring in both a systemic and intestinal manner in ALS patients. Limitations of this study include small sample sizes and the presence of various confounding factors that can include diet and secondary disease effects.

A 2021 study (Niccolai et al., 2021) found the differences in 15 bacterial species between the ALS patients and healthy controls. This included butyrate-prodiucing bacteria *Roseburia intestinalis* and *Eubacterium rectale*. The total relative abundance of several butyrate producers was significantly lower in ALS patients compared to healthy controls. *Bilophila (unclassified)*, *Clostridiaceae bacterium JC1118*, *Coprobacter fastidiosus*, *Eubacterium eligens*, and *Ruminococcus sp 5 1 39 BFAA* was lower in ALS patients compared to controls, while the relative abundance of *Escherichia (unclassified)* and *Streptocossus salivarius* was higher in ALS patients. To avoid the potential changes that occur in late term ALS, they compared the samples from PALS that were collected within one year after diagnosis, the relative abundance of both *Roseburia intestinalis* and *Eubacterium rectale* were significantly lower in the ALS patients compared to healthy controls. The levels of total butyrate-producing bacteria were lower in the PALS patients compared to healthy controls, but the difference was not statistically significant. In patients that were not using Riluzole at the time of sample collection, they found that the results were similar – Roseburia intestinalis, Eubacterium rectale, and total abundance of butyrate-producing bacteria were lower in these ALS patients compared to healthy controls. The results were the same when excluding ALS patients that were using a gastrostomy tube. Overall, they found reduced levels of anti-inflammatory SCFA-producing bacteria in patients with ALS. A limitation of this study could be that patients unintentionally changed their diet after developing ALS, therefore altering the gut microbiota composition. Dietary differences were unfortunately not able to be accounted for. This study further indicates that gut microbiome could act as an integral component in the pathophysiology of ALS.

A 2022 study (Zhang et al., 2022) investigated the connection between the gut microbiota and ALS in a GWAS of ALS that involved 20,806 patients and 59,804 control individuals. They found that *OTU10032 unclassified Enterobacteriaceae* (2 independent SNPs) and *unclassified Acidaminococcaceae* (4 uncorrelated SNPs) were associated with a higher risk of ALS. Gamma-glutamyl amino acids demonstrated possible negative effects of the risk of developing ALS. Gamma-glutamylphenylalanine specifically displayed an increased risk of developing ALS that was significant. 1-arachidonoyl-GPI and 3-methyl-2-oxobutyrate, two metabolites, were connected with an increased risk of developing ALS. An increase in the levels of 4-acetamidobutanoate may lower the risk of developing ALS. Additionally, an increase in the relative abundance of *OTU4607_Sutterella* was associated with genetically predicted ALS. An increase in the relative abundance of *Lactobacillalesorder* was associated with genetically predicted ALS. This study showed that a bidirectional relationship between the gut microbiota and ALS. Limitations of this study include weak instrument bias and population stratification, though this bias was somewhat reduced by restricting the dataset to individuals of European ancestry.

A 2022 study (Hertzberg et al., 2022) analyzed the gut microbiota of ALS patients while using the patients’ spouses as the healthy controls. The authors found that the ALS patients did not have any butanoate metabolic pathway enzymes. They also displayed lower amounts of enzymes that are utilized in various metabolic pathways. These include pathways involving response regulators and carbon metabolism pathways. When comparing the 25 stool and inflammatory markers, including IL-2, IL-4, tumor necrosis factor, and interferon, there were no statistically significant differences between the ALS patients and the healthy controls. Additionally, ALS patients did not have amplicon sequence variants of the genus *Prevotella*, specifically *P. timonensis*, compared to the healthy controls. This specific result was consistent with other studies that suggest that gut microbiome dysbiosis is associated with neurodegenerative diseases. The authors suggested that the difference in P. timonensis in ALS patients versus the controls may have contributed to the lack of differences in pro-inflammatory cytokine levels. In contrast to previous studies, they did not find a lack of butyrate-producing microbes. However, they found a decrease in the enzymes associated with butyrate metabolism, which is consistent with previous studies, suggesting an association between gut butyrate availability and progression of ALS. Further studies looking at this association would be needed. Limitations of this study include the small sample size and the lack of diet information. To overcome the small sample size, larger scaled studies would need to be conducted.

In a 2019 study (Zhai et al., 2019) looking at 8 patients with ALS and 8 healthy controls, they found that the richness and evenness of bacterial and archaeal communities of healthy individuals were healthier than those of the ALS patients. They also found that the average relative abundance of *Firmicutes* in ALS patients was 4.7% higher than the abundance in healthy controls. On the class level, the relative abundance of *Negativicutes* and *Bacili* were decreased compared to healthy controls. The relative abundance of phylum Euryarchaeota, class Methanobacteria, and genus *Methanobrevibacter* were all significantly increased in ALS patients compared to the healthy controls. While there were no significant differences in metabolites between ALS patients and healthy controls, there was an increase in SCFA, NO_2-_N/NO_3_N, and γ-aminobutyric acid in ALS patients compared to healthy controls. The results of this study suggested that the biodiversity and composition of intestinal microflora in ALS patients is of lower quality than that of controls. The increase in the Firmicutes/Bacteroidetes ratio and *Methanobrevibacter* indicates that there is a gut flora composition imbalance in patients with ALS. One limitation of this study was that metagenomic sequencing was not performed in order to analyze microbial function variation. Another limitation would be the need for microbial community acclimation in order to better understand the composition of the microbiota in ALS patients.

In a 2020 study (Ngo et al., 2020) that looked at the relationship of the fecal microbiota and prognosis of ALS, they found that ALS patients with a higher richness and evenness of their fecal microbiome had a decreased survival from their onset of symptoms than the patients who had less richness and evenness. There was no significant difference in *Proteobacteria* between ALS patients and controls. There were also no significant differences in the proportional abundance in the two most abundant phyla, *Firmicutes* and *Bacteriodetes*, between ALS patients and controls. Overall, they found the fecal microbiota in a group of ALS patients is not significantly different than those of healthy controls. They believe that clinical and metabolic features of ALS are not connected with the fecal microbiome composition. However, the diversity and richness of the fecal microbiome may be associated with an increased risk of an earlier death. The results suggest that the fecal microbiota of ALS patients does not differ from those of healthy individuals. One limitation of this study is that they included both patients who were newly diagnosed and patients who had already progressed and were attending clinics. Other limitations would be a small sample size and the amplifying of variability due to the patients’ dietary habits.

A potential proxy for the effect of the gut microbiome on disease is the effect of oral antibiotics which significantly modify the balance of gut microbial species. A 2019 study demonstrated that any use of antibiotics, especially repeated use, was associated with increased risk of ALS in the native Swedish population (2006-2013 period) (Sun et al., 2019). Similarly, SOD1^G93A^ mice repeatedly exposed to antibiotics develop a more severe motor phenotype and increased motor neuron loss (Blacher et al., 2019),

We summarized the studies on gut microbiome in ALS patients in **Table 4**. These results suggested novel roles of intestinal microbiome in the development of ALS and the potential benefits of decreasing the number of pathogens and increasing probiotics. The challenge of early studies of microbiome in ALS lies in the following aspects: 1) limited sample size; 2) preliminary data analysis; and 3) reluctance from neurologists to pay attention the intestinal changes in the early stage of ALS. In the earlier time of microbiome research, a F/B ratio was used. Although it indicated the evidence of dysbiosis in patients with ALS. The F/B ratio only reflect the relative abundance of bacteria at the phylum level and may miss the changes at the species, thus, it is not a reliable readout for the in-depth analysis and advanced studies of microbiome data.

**Table 4:** Microbiome studies in patients with ALS.

Study	Key Bacterial changes	Sample size	Methods	Reference
Microbial diversity in ALS	*Bacteroidetes*, *bacteroidia*, *bacteroidales*, and *dorea* were significantly higher in ALS patients. A significant decrease of *Oscillibacter*, *Anaerostipes*, and *Lachnospiraceae* in ALS patients compared to controls.	Six ALS patients and five healthy people without ALS	Extracted genomic DNA and high-throughput sequencing.	https://pubmed.ncbi.nlm.nih.gov/27703453/
The alteration of gut microbiome and metabolism in ALS patients	Increases in *Bacteriodetes*, *Kineothrix*, *Parabacteroides*, *Odoribacter*, *Sporobacter*, *Eisenbergiella*, *Mannheimia*, *Anaerotruncus*, unclassified *Porphyromonadaceae* in ALS patients. Significant reduction in *Firmicutes* and *Megamonas* in ALS patients compared to control patients.	20 patients with probable or definite ALS and 20 healthy controls	16s rRNA sequencing; Shotgun sequencing (consisting of 10 ALS patients and 10 controls; Metabolomics analysis.	https://pubmed.ncbi.nlm.nih.gov/32747678/
The fecal microbiome of ALS patients	Different proportions of *Ruminococcaceae*	25 ALS patients (2 familial, 23 sporadic) and 32 controls	Fecal samples collected, extraction of nucleic acids, qRT-PCR, 16S rRNA sequencing analysis	https://pubmed.ncbi.nlm.nih.gov/29065369/
Gut inflammation and dysbiosis	A low *Firmicutes/Bacteroidetes* ratio and low *Ruminococcus* spp. in ALS patients 1,3, and 5, low *Clostridium* spp. and *Roseburia* spp. in patient 1, high *Bacteroides-Prevotella*, *Odoribacter* spp. *Barnesiella* spp., and *Bacteroides vulgatus* in patient 5, and high *Bacteroides vulgatus* in patient 3	5 ALS patients and 96 healthy individuals	Collected stool samples, commensal bacteria PCR, bacterial and mycological cultures, mass spectrometry, enzyme immunoassay	https://pubmed.ncbi.nlm.nih.gov/28947596/
A prospective longitudinal study on the microbiota composition in ALS	longitudinal study addressing the microbiota composition in ALS patients and the role of a probiotic supplementation on the gut microbiota and disease progression. Lower *Clostridium* and higher *E. coli and* Enterobacteriaceae were detected in ALS patients. Members of the Cyanobacteria phylum were significantly higher in ALS patients. The control group showed a higher relative abundance of Veillonellaceae, Promicromonosporaceae, and Peptostreptococcaceae.	50 ALS patients and 50 matched controls	DNA extraction from fecal samples, quantitative PCR, PCR-DGGE	(Di Gioia et al., 2020) https://pubmed.ncbi.nlm.nih.gov/32546239/
Assessment of bidirectional relationships between 98 genera of the human gut microbiota and ALS	*OTU10032 unclassified Enterobacteriaceae* was associated with a higher risk of ALS. *Unclassified Acidaminococcaceae* was associated with a higher risk of ALS. Gamma-glutamylphenylalanine showed a significantly increased risk of ALS. Two metabolites, 1-arachidonoyl-GPI and 3-methyl-2-oxobutyrate, were associated with a higher risk of ALS. A genetically predicted increase in the levels of 4-acetamidobutanoate may lower the risk of ALS. Genetically predicted ALS was associated with an increase in the relative abundance of *OTU4607_Sutterella* and *Lactobacillales_order*.	98 genera of gut microbiota, GWAS of ALS involving 20,806 patients and 59,804 controls of European ancestry	Genome wide association study, Mendelian randomization analysis, identification of independently significant SNPs	https://pubmed.ncbi.nlm.nih.gov/34979977/
The human gut microbiota in people with ALS	There was a significantly lower abundance of both *Roseburia intestinalis* and *Eubacterium rectale* in patients in their first year of diagnosis compared to healthy controls. These results were similar to patients who were not taking Riluzole at the time of sample collection, and patients not using a gastrostomy tube. The relative abundance of the family *Lachnospiraceae* was lower in ALS patients compared to healthy controls, but the results were not statistically significant. The total relative abundance of the eight dominant butyrate producers was significantly lower in ALS patients compared to controls. A higher abundance of butyrate-producing bacteria was associated with a significantly lower risk of ALS.	66 patients with ALS, 61 healthy controls, 12 neurodegenerative controls	Clinical information was collected, stool sample self-collected, DNA and RNA extraction, metagenomic shotgun sequencing and profiling, ribosomal sequencing and profiling	https://pubmed.ncbi.nlm.nih.gov/33135936/
The Gut Microbiota-Immunity Axis in ALS: A role in deciphering disease heterogeneity	ALS patients displayed lower amounts of cytokines IL-15, IL-8, MCP-1 and VEGF-A compared to healthy controls ALS patients with a classical phenotype showed a lower microbial diversity compared to others	19 ALS patients and 9 healthy family caregivers matched for sex and closely aligned age	Fecal samples collected, tested 30 cytokines using Luminex MAGPIX detection system, qualitative and quantitative evaluation of SCFAs, genomic DNA extraction and sequencing	https://pubmed.ncbi.nlm.nih.gov/34209688/
Progression and Survival of patients with motor neuron disease relative to their fecal microbiota	There were no significant differences in the distribution of bacteria, Proteobacteria, and F/B between ALS patients and controls. ALS patients with greater richness and evenness of their fecal microbiome had worse survival from the onset of their symptoms than ALS patients who had a decreased richness and evenness.	64 patients with ALS and 74 controls (spouses, friends, or family members of ALS patients)	Measurements of whole-body composition and resting energy expenditure, fecal sample collection, DNA extraction, and 16s rRNA amplicon sequencing	https://pubmed.ncbi.nlm.nih.gov/32643435/
Bacterial and archaeal communities, butyrate concentration analyses in ALS patients	The richness and evenness of bacterial and archaeal communities of healthy controls was “healthier” than those found in ALS patients. The average relative abundance of phylum *Firmicutes* in ALS patients was 4.7% higher than the relative abundance in controls. The relative abundance of *Negativicutes* and *Bacili* on the class levels were decreased in ALS patients compared to controls. The relative abundance of phylum Euryarchaeota, class Methanobacteria and genus *Methanobrevibacter* was significantly increased in ALS patients. There was no significant difference in fecal butyrate concentration between patients with ALS and healthy controls.	8 patients with ALS and 8 healthy individuals	Fecal samples collected, PCR amplification, enzyme linked immunosorbent assay (ELISA), fecal metabolites, endotoxin, short-chain fatty acids, NO2-N/NO3-N, and γ-aminobutyric acid, were evaluated by spectrophotometry	https://pubmed.ncbi.nlm.nih.gov/31306225/
Potential roles of gut microbiome and metabolite nicotinamide in modulating ALS	Confirm the dysbiosis and aberrant metabolism of nicotinamide in the ALS patients. Significant alterations in key molecules of the tryptophan–nicotinamide metabolic pathway in sera of patients with ALS—among them indoleacetate, kynurenine, serotonin and circulating nicotinamide.	37 human ALS patients and 29 age-matched controls	For human patients, collected their stool samples and sequenced their gut microbiome metagenomes, targeted serum metabolomics	https://pubmed.ncbi.nlm.nih.gov/31330533/
Gut microbiome differences between ALS patients and spouse controls	ALS patients lacked ASVs of the *Prevotella* genus compared to their spouses, particularly *P. timonensis.* There was no difference in stool and plasma inflammatory biomarkers between ALS patients and their spouses. ALS patients had a difference in beta diversity from their spouses. Butanoate metabolic pathway enzymes were missing in patients with ALS.	10 individuals with ALS and their spouses and 10 healthy couples	Rectal and blood sample collection, DNA extractions and 16s rRNA gene sequencing, measurement of inflammatory markers, enzyme linked immunosorbent assay (ELISA) bioinformatics	https://pubmed.ncbi.nlm.nih.gov/33818222/
Potential Role of Gut Microbiota in ALS Pathogenesis and Possible Novel Therapeutic Strategies	There was a lower DNA concentration in ALS patients compared to the healthy controls. There was a low abundance of *Clostridium* and yeast and a high abundance of *E. coli* and *Enterobacteria* in ALS patients compared to controls. There was a clear cluster division between the bacterial profiles of ALS patients compared to the healthy controls. There was no association between yeast profiles and the presence or absence of ALS.	50 ALS patients and 50 healthy controls, matched for sex, age, and origin	Fecal sample collection, total genomic DNA extraction, PCR-denaturing gradient gel electrophoresis analysis, quantitative pCR, double-blind treatment of ALS patients	https://pubmed.ncbi.nlm.nih.gov/29782468/

## Intestinal Inflammation in ALS

There is abundant evidence many substances involved in the promotion of inflammatory processes are present in the CNS of patients. Previous reviews have discussed the role of inflammation in ALS and the possibility of treating ALS by immune modulation (McCombe and Henderson, 2011; Kwon and Koh, 2020). Dysregulation of the peripheral immune system and reduced levels of regulatory T cells are associated with disease progression. Monocytes are activated in ALS and dendritic cells play a role in pathogenesis. In the brain and spinal cord of patients with ALS, microglia respond to injury in a beneficial way, but chronic microglial activation is thought to contribute to the pathogenesis of the neurodegenerative disease. There is activation of microglia (‘neuroinflammation’) in ALS. Microglia can have opposite roles at different times. However, the local inflammation in other organs, e.g GI, was less explored in the ALS research.

Intestinal Paneth cells in the small intestine produce most of the antimicrobial peptides (AMPs) and play an important role in regulating the innate immunity and inflammation (Deretic et al., 2008; Lu et al., 2021). The Paneth cell is a unique that can sense commensals and secrete AMPs, thereby playing critical roles in the maintenance of homeostasis at the intestinal-microbial interface. There was a decreased number of normal Paneth cells in the small intestine of SOD1^G93A^ mice, whereas abnormal Paneth cells were significantly increased in SOD1^G93A^ mice. There was a decreased amount of the AMP defensin 5 alpha in the intestine of the 3-month-old SOD1^G93A^ mice with disease symptoms. There was a significant reduction in lysozyme 1 in the G93A intestine, but lysozyme 2 was not altered. There were no pathological changes in the small intestine of G93A mice. There were increased serum IL-17 levels in the young G93A mice at 2 months of age. IL 17 was increased in the intestine of G93A mice, suggesting a preinflammatory state in ALS mice before disease onset. There were higher FITC readings that indicated higher permeability of the intestine. Our study has demonstrated Paneth cell dysfunction and inflammation in the intestine of G93A mice. The intestinal inflammation may occur earlier than the systemic inflammation.

A recent study (Figueroa-Romero et al., 2019) in the SOD1^G93A^ model showed that dysbiosis was observed in the 37 day-old mice. Moderate differences in the peripheral system beginning at 90 days, with elevated CD8T cells in the spleen, and then at 120 days with CD4T cell counts in peripheral blood. The CNS inflammation occurs during late-stage disease in the SOD1^G93A^ mice. This study provides a roadmap to the chronological changes that occur in the intestinal microbiome and immune system relative to disease onset and progression in the SOD1^G93A^ mouse model. It further supports the critical role of microbiome in the early stage of ALS pathogenesis (Zhang et al., 2021). Limitations of this study include that there may have been some variation introduced by a separate cohort of 120-day-old SOD1 G93A mice. Additionally, the number of males versus females was not always equal during the course of the study.

A previous study (Zhang et al., 2009) reported that the level of plasma LPS showed a significant increase, correlating with blood monocyte/macrophage activation in sALS groups, especially those with advanced sALS disease (Zhang et al., 2009). A report (Tortelli et al., 2020) studied a panel of five cytokines (IL-2, IL-6, IL-10, IFN-gamma, and TNF-alpha) in plasma in ALS (79 patients with ALS and 79 age- and sex-matched healthy controls). All the five cytokines were significantly increased in plasma samples of patients compared with controls, with IL-6 having the highest median concentration (10.11 pg/ml) in the ALS group. Furthermore, IL-6 was the plasma cytokine with the highest discrimination ability between patients and controls according to the receiver operating characteristic analysis. The elevated inflammatory cytokines in ALS support a possible role of inflammation in disease progression. Recent animal experimental studies (Butovsky et al., 2012) have tried to modulate of inflammatory monocytes with microRNAs in ALS mice.

In the animal model of C9orf72 study (Burberry et al., 2020), transplantation of pro-survival intestinal microbiome in the C9orf72^Harvard^ resulted in a significant decrease of inflammatory and autoimmune phenotypes, compared with transplantation of pro-inflammatory microbiome. These results suggested that signals from gut microbiota can help maintain the suppression of certain inflammatory and autoimmune phenotypes. *Helicobacter* spp. was found in both pro-inflammatory environments, but not in pro-survival environments. The abundance of 62 of 301 bacterial species were significantly changed when comparing the two pro-survival environments to the two-pro-inflammatory environments.

Gut microbes can influence the innate and adaptive immune systems, including immune cells in the gut and the systemic immunity.

Neuroinflammation contributes to the pathogenesis of ALS through various mechanisms including enhanced reactive oxygen species (ROS), activation of innate immune systems, increased proinflammatory cytokines, and infiltration of immune cells. Dysbiosis leads to an inflammatory state and altered gut microbiota could influence the immune system in ALS. Moreover, the nutrition has a direct impact on gut microbiome, which shapes local intestinal immune responses and in turn affects even autoimmune responses. Conjugated linoleic acid (CLA), a naturally occurring fatty acid in meat and dairy products of ruminants. A recent study (Fleck et al., 2021) identified CLA as a potent modulator of the gut–CNS axis by targeting myeloid cells in the intestine, which in turn controlled encephalitogenic T-cell responses in mouse model of multiple sclerosis and a pilot human study. However, microbiome, nutrients, immune cells, and cytokines in early disease pathology of human ALS are still unclear. The precise role of inflammation in various organs, include GI, in ALS needs to be further investigated on larger samples and with more mechanistic studies.

## Targeting Microbiome and Metabolites in ALS Treatment

Significant alterations in the levels of various metabolites were identified in the sera of ALS patients. Bacterial product butyrate is beneficial and slows down the progression of ALS, based on our studies in the SOD1^G93A^ mice (Zhang et al., 2017; Zhang et al., 2021). There is a trial (Paganoni et al., 2020; Turnbull, 2020) using sodium phenylbutyrate-taurursodiol in ALS patients. It reported Sodium phenylbutyrate-taurursodiol resulted in slower functional decline than placebo as measured by the ALSFRS-R score over a period of 24 weeks.

There were alterations in the levels of key components of the tryptophan-nicotinamide (NAM) pathway in some ALS patients. Specifically, the levels of the metabolite NAM in the CSF of 14 patients with ALS were significantly lower than the levels in 17 healthy controls. These results suggested that ALS does interact in some manner with the intestinal microbiota of patients and creates a cycle in which the increase in pathogens and decrease in probiotic organisms in the intestines of ALS patients can upregulate or downregulate the production of NO, GABA, and SCFAs, increasing the pathogenesis of ALS which furthers the imbalance of the intestinal microbiota. The gut microbiota produces metabolites of tryptophan that play a role in regulating astrocyte and microglial activation. Combined, these findings suggest a possible role for microbial metabolites in the control of neuroinflammation during ALS disease progression. Targeting the gut-brain axis could modify the neuronal function (Huang et al., 2019; Kang et al., 2019) and possibly contribute to some of the non-motor cognitive and behavioral symptoms in ALS, but this would require further study.

Riluzole is FDA proved disease-modifying treatment for ALS and has the potential to extend life by only a few months (Miller et al., 2012). Interestingly, Riluzole was significantly metabolized by 40 of the bacteria, based on a 2019 study of on drug metabolism by microbiome (Zimmermann et al., 2019). Many of these bacteria are known to vary in prevalence in the human population. However, the role of the microbiome has not been linked to the drug metabolism and management of ALS treatment.

The use of proton pump inhibitor (PPI) drugs has been associated with a decrease in gastric pH, leading to gastrointestinal dysbiosis (Minalyan et al., 2017). It has also been suggested that PPI use may be related to an increased risk of neurodegenerative diseases (Erber et al., 2020). A retrospective cohort study conducted in Austria reported that 97.5% of ALS patients use other drugs together with riluzole, including PPI and centrally acting muscle relaxants (CAMR). A significant trend in PPI in reducing the survival of ALS was noted; however, after statistical corrections were applied, this effect was no longer observed. On the other hand, the use of CAMR in association with riluzole showed a beneficial effect on the survival of these patients (Cetin et al., 2015). In a case-control study conducted with 2,484 ALS patients in Sweden, no correlation between PPI use and risk of ALS was observed in a lag window of 1-3 years (Cetin et al., 2020). Thus, it is still lacking evidence to support the detrimental effect of PPI in ALS; nevertheless, 50% of ALS patients report using PPI, raising the importance of further investigations to better understand this correlation.

The increase in the abundance of *Rikenellaceae* was significant in ALS patients who underwent a 6-month probiotic treatment administered daily (Di Gioia et al., 2020). The probiotic formulation is a mixture of five lactic acid bacteria: *Streptococcus thermophilus* ST10–DSM 25246, *Lactobacillus fermentum* LF10–DSM 19187, and *Lactobacillus delbrueckii* subsp. *delbrueckii* LDD01–DSM 22106, *Lactobacillus plantarum* LP01–LMG P-21021, and *Lactobacillus salivarius* LS03–DSM 22776. No adverse events attributed to probiotic supplementation. The significant increase in *Cyanobacteria* at the phylum, family, and genus level in ALS patients compared to controls, suggesting that cyanobacteria play a critical role in the pathogenesis of neurodegenerative diseases. The abundance of cyanobacteria decreased overtime in both the probiotic and placebo groups, although the difference was not significant.

Fecal microbiota transplantation (FMT) could improve gastrointestinal and behavioral symptoms in neurological diseases (Kang et al., 2017; Kang et al., 2019). Parkinson’s disease patients after one-week treatment of FMT improved constipation and motor symptoms such as leg tremors. The tremors recurred 2 months after FMT, whereas constipation was relieved even after 3 months (Huang et al., 2019). Intestinal bacteria as an external trigger could explain the rare cases of ALS in spouses or in some clusters (Sabel et al., 2003). To reconstruct the intestinal microbiome, FMT has been used to transfer the gut microbiota from healthy individuals to ALS patients (Xu et al., 2021). ClinicalTrials.gov (https://clinicaltrials.gov/ct2/show/NCT03766321) has an ongoing FMT trial for a year with 42 ALS patients, at an early stage (28 FMT-treated patients *vs.* 14 controls). This will promote a further understanding of microbiota restoration and GI function.

## Diet, Microbiome, and ALS

Many factors are involved in the gut microbiota composition and function, including genetics, gender, age, geographic location, lifestyle, and diet (Ballan et al., 2020; de Vos et al., 2022). Also, the gut microbiota is responsible for metabolization of compounds from the diet producing several metabolites (Ballan et al., 2020; de Vos et al., 2022). As a consequence, different gut microbiota compositions will result in different metabolite profiles, that will interact with cell receptors, having a direct impact on different signaling pathways and on the host health (de Vos et al., 2022).

Diet is associated with the ALS risk, onset, and progression, providing potential modifiers of disease (Pape and Grose, 2020). Two mutant SOD1(G86R and G93A) mice exhibited an altered metabolic status (Dupuis et al., 2004). This study provided evidence that transgenic ALS mice suffer from a dramatic defect in energy homeostasis, likely linked to a hypermetabolism mainly of muscular origin. High-energy-diet improved the altered metabolic phenotype of G86R mice and delays disease onset (Dupuis et al., 2004). Several epidemiological studies have identified diets that positively affect ALS patients, including various high-calorie fat or sugar-based diet, summarized in a recent review (Pape and Grose, 2020).

Gluten-induced autoimmunity was speculated to trigger ALS. However, the data supporting this link are weak (Group, 2016). Leptin is an adipokine involved in food intake regulation and energy balance. Lower level of circulating leptin is associated with fat mass loss, and thus, body weight loss in ALS. Moreover, it has been suggested that fat mass loss occurs prior to muscle atrophy. Thus, leptin could be a potential biomarker of adipose tissue wasting in the early stages of ALS. The appetite-stimulating hormone ghrelin also seems to be downregulated in an ALS mice model, compared with their WT controls, contributing to lower food intake and body weight loss (Ferrer-Donato et al., 2021).

Earlier studies suggested that polyphenols (e.g., resveratrol, curcumin, epigallocatechin gallate, quercetin, and phenolic acids), which can be found in fruits, vegetables, coffee, tea, and whole grains, may have a promising neuroprotective effect in ALS. It was observed, *in vivo* and *in vitro*, that these bioactive compounds may have the potential to regulate mitochondrial biogenesis, improve energy metabolism, reduce toxic protein aggregation, reduce microglia and astrocytes inflammation, and improve motor functions and survival (Solanki et al., 2015; Novak et al., 2021).

Resveratrol, an antioxidant compound found in grapes, has been widely studied due to its neuroprotective properties. Resveratrol may reduce the *in vitro* neurotoxicity of cerebrospinal fluid (CSF) from ALS patients, preventing neuronal loss and improving Ca^2+^ homeostasis, which seems to be related to the antioxidant capacity of resveratrol. Curiously, co-incubation with riluzole inhibited this protective effect (Yanez et al., 2011). In fact, Ca^2+^ dyshomeostasis is related to impaired autophagy mechanisms and toxic protein aggregation in neurodegenerative disorders, including ALS (Tedeschi et al., 2019). Therapeutic interventions aiming to modulate autophagy pathways seemed to be an interesting approach to reduce protein aggregates, mainly in the early stages of ALS (Cipolat Mis et al., 2016).

A prospective study reported that probiotics may promote beneficial effects in ALS patients. The administration of probiotics to ALS patients increased the relative abundance of groups related to propionate and butyrate production in gut microbiota, which may improve energy provision. Also, it has been speculated a possible metabolization of neurotoxic compounds (Di Gioia et al., 2020).

In our previous work, we observed the administration of butyrate, a beneficial microbial metabolite, delayed the disease progress and significantly extended the survival time of the SOD1 ^G93A^ mice and prolonged the life span by 38 days on average (Zhang et al., 2017). Butyrate treatment improved the gut microbiome and restored the Paneth cells and the signaling of lysozyme 1 and anti-microbial peptide defensin 5 alpha in the SOD1 ^G93A^ mice. Meanwhile, the intestinal protein aggregation of SOD1^G93A^ was significantly decreased by butyrate treatment (Zhang et al., 2017). Furthermore, butyrate treatment was able to enhance healthy metabolites by longitudinal untargeted metabolomic analysis in ALS (Destiny Ogbu and Claud, 2022).

Nicotinamide showed slightly delay of the disease progression in the ALS SOD1^G93A^ mice (Blacher et al., 2019). The survival curve showed only several days difference between the SOD1^G93A^ mice with or without nicotinamide treatment. Aberrant metabolism of nicotinamide was observed in the sera of patients with ALS. There was a trial of nicotinamide/pterostilbene supplement in ALS (https://clinicaltrials.gov/ct2/show/NCT04562831). The results have not been reported yet.

Emerging evidence support the roles of gut-neuron-microbiome in various human diseases (Ogbu et al., 2020; Fang et al., 2020). For examples, in a human pilot study in patients with multiple sclerosis, dietary conjugated linoleic acid-supplementation for 6 months significantly enhanced the anti-inflammatory profiles and functional signatures of circulating myeloid cells (Fleck et al., 2021). To move forward, we hypothesize that dietary interventions with functional foods (e.g., prebiotics, probiotics, polyphenols) or the administration of probiotic metabolites (e.g., butyrate) aiming the gut microbiota modulation may enhance intestinal barrier functions and autophagy regulation, restore healthy host-microbial interactions, and reduce toxic protein aggregation and inflammation, thus, delaying the progression of ALS.

## Conclusion and Future Directions

ALS is a progressive neurodegenerative disorder involving brain and spinal cord motor neuron death resulting in weakness and wasting of musculature and leaning inexorably to death. There are GI abnormalities involved liver, intestine, and pancreas in ALS. Subclinical gastrointestinal motor dysfunction, delayed colonic transit time, delayed gastric emptying an increased prevalence of constipation and anal sphincter abnormalities have been noted, but the mechanism of these issues is unclear. In various mouse models of ALS, the altered microbiome and GI issues are also observed. However, factors such as changes of diet, GI issues, inflammation, and infection were not well considered for the ALS diagnosis.

Emerging evidence has demonstrated the novel roles of microbiome and metabolites in the early stage of disease development and progression, suggesting the potential biomarkers (Chatterjee et al., 2020; Donatti et al., 2020). SALS and fALS forms of ALS manifest similar pathological and clinical phenotypes, suggesting that different initiating causes lead to a mechanistically similar neurodegenerative pathway. ALS patients have abnormal GI systems, elevated intestinal inflammation and dysbiosis. The ENS and smooth muscle automatism are unable to modulate the motor functions of the digestive tract, which provide an anatomical explanation for these clinical manifestations. Studies on microbiome changes with pro-inflammatory serum cytokines and LPS in patients will help with the early diagnosis of the disease. To treat gut microbiota dysbiosis through microbiota restoration would have the potential to interfere and slow ALS progression (Mandrioli et al., 2019). The therapeutic methods to target microbiome and intestinal functions, e.g, FMT, prebiotics, and probiotics, will help both SALS and FALS patients. ALS studies should be performed by considering diet, microbiome, lifestyle, and gender difference. Studies that examine the microbiome together with intestinal pathogenesis will help to determine when, where, and whether microbiome and metabolites are critical to disease progression of ALS. Understanding the pathogenesis of ALS GI will provide innovative strategies for accurate diagnosis and better treatment for this challenging disease.

## Author Contributions

SM and JS performed literature search and detailed analyses and summary of related literature, prepared the draft text. CB contributed to literature search of ALS mouse models, nutrition, and the draft text. JS designed the study/project, obtained funds, summarize literature, and directed the project. All authors contributed to the article and approved the submitted version.

## Funding

We would like to acknowledge the VA Merit Award 1 I01BX004824-01, the NIDDK/National Institutes of Health grant R01 DK105118, and R01DK114126 to JS. The study sponsors play no role in the study design, data collection, analysis, and interpretation of data. The contents do not represent the views of the United States Department of Veterans Affairs or the United States Government.

## Conflict of Interest

The authors declare that the research was conducted in the absence of any commercial or financial relationships that could be construed as a potential conflict of interest.

## Publisher’s Note

All claims expressed in this article are solely those of the authors and do not necessarily represent those of their affiliated organizations, or those of the publisher, the editors and the reviewers. Any product that may be evaluated in this article, or claim that may be made by its manufacturer, is not guaranteed or endorsed by the publisher.
